# Phenolic content, antioxidant effect and cytotoxic activity of *Leea indica* leaves

**DOI:** 10.1186/1472-6882-12-128

**Published:** 2012-08-17

**Authors:** Nidyaletchmy Subba Reddy, Suerialoasan Navanesan, Saravana Kumar Sinniah, Norhanom Abdul Wahab, Kae Shin Sim

**Affiliations:** 1 Institutional address: Institute of Biological Sciences, Faculty of Science, University of Malaya, 50603 Kuala Lumpur, Malaysia; 2 Institutional address: Biology Division, Centre for Foundation Studies In Science, University of Malaya, 50603, Kuala Lumpur, Malaysia

**Keywords:** *Leea indica*, Vitaceae, Antioxidant, Colon cancer cells, Phenolic content

## Abstract

**Background:**

The leaves of *Leea indica* (Vitaceae), commonly known as ‘Huo Tong Shu’ in Malaysia, have been traditionally used as natural remedy in folk medicine by the locals. The current study reports the outcome of antioxidant and cytotoxic investigation of *L. indica* leaves. To the best of our knowledge, this is the first report of *L. indica* leaf crude ethanol and its fractionated extracts (hexane, ethyl acetate and water) for evaluation of total phenolic content, antioxidant effect and cytotoxic activity against colon cancer cell lines.

**Methods:**

In the present study, *L. indica* leaf crude ethanol and its fractionated extracts (hexane, ethyl acetate and water) were firstly prepared prior to phenolic content, antioxidant effect and cytotoxic activity assessment. Folin-Ciocalteau’s method was used for the measurement of total phenolic content of the extracts. The antioxidant activity was measured by employing three different established testing systems, such as scavenging activity on DPPH (1,1-diphenyl-2-picrylhydrazyl) radicals, reducing power assay and SOD (superoxide dismutase) activity assay. The cytotoxic activity of the extracts were evaluated against three colon cancer cell lines with varying molecular characteristics (HT-29, HCT-15 and HCT-116) by MTT [3-(4,5-dimethylthiazol-2-yl)-2,5-diphenyltetrazolium bromide] assay.

**Results:**

The total phenolic content and antioxidant capabilities differed significantly among the *L. indica* leaf extracts. A strong correlation between total phenolic content and antioxidant properties was found, indicating that phenolic compounds are the major contributor to the antioxidant properties of these extracts. Among the crude ethanol and its fractionated extracts, fractionated water extract showed significantly the highest total phenolic content and strongest antioxidant effect in all the antioxidant testing systems employed in this study. All the four extracts exert no damage to the selected colon cancer cells.

**Conclusions:**

The data obtained in these testing systems clearly establish the antioxidant potency of the fractionated water extract of *L. indica* leaves. Additional studies should be carried out to isolate and identify the bioactive compounds in the fractionated water extract, in order to provide more convincing evidence.

## Background

At present, naturally derived products play an important role as source of medicine. Many pharmaceutical agents have been discovered by screening natural products from plants, based on ethnopharmacological data which provides a substantially increased chance of finding active plants relative to a random approach.

*Leea indica* (Vitaceae), commonly known as ‘Huo Tong Shu’ in Malaysia, have been traditionally used as natural remedy in folk medicine by the locals. It is a perennial shrub which can be found in tropical and subtropical countries, such as Thailand, Malaysia, India and China. The leaves and roots of *L. indica* are traditionally used for the treatment of cancer, diabetes, diarrhea, dysentery, spasm and skin diseases [1,2]. The leaves are generally consumed by locals either raw or taken as a concoction brewed from fresh leaves. The whole plant is also used as remedy for the relief of headache, body pains and skin complains [3].

There are limited phytochemical studies reported on *L. indica* leaves [4-6] and essential oil of flowers [7]. To our knowledge, although the leaves of *L. indica* is reported to be used in a large number of Malaysian traditional medicine preparations, there is not much recorded data on biological studies of *L. indica* leaves. An investigation by Saha et al. [8] reported that the crude methanol extract from the whole plant of *L. indica* showed high antioxidant and nitric oxide inhibitory activities, by employing FTC (ferric thiocyanate), TBA (thiobarbituric acid), DPPH free radical scavenging methods and Griess assay. A later report by Temkitthawaon et al. [9] indicated that the crude ethanol extract of *L. indica* roots showed potent phosphodiesterase inhibitory activity. However, a report by Nurhanan et al. [10] stated that the crude methanol extracts of leaf, stem and bark of *L. indica* did not showed any anti-proliferative activities against the breast cancer cell lines. Additionally, the essential oil of *L. indica* flowers showed only moderate antibacterial activity against the tested bacteria [7]. More recent investigations by Hsiung et al. [11] and Wong et al. [12] reported that the ethyl acetate fraction of *L. indica* leaves and the mollic acid arabinose isolated from it induced growth-inhibitory effect and apoptosis in Ca Ski human cervical cancer cells.

The current study aimed to investigate the total phenolic content, antioxidant effect and cytotoxic activity of *L. indica* leaves. The antioxidant potency of *L. indica* leaves have been investigated, employing three different established *in vitro* testing systems, such as scavenging activity on DPPH radicals, reducing power assay and superoxide dismutase (SOD) activity assay. The total phenolic content of the leaf extracts was also accessed by Folin-Ciocalteau’s method. To our knowledge, there is no antioxidant study reported for *L. indica* leaves. Thus, the antioxidant activity of *L. indica* leaves was evaluated as it has not been determined previously.

In view of the traditional usage of *L. indica* in cancer-related diseases and the investigation by Hsiung et al. [11] and Wong et al. [4,12] which indicated the potential use of *L. indica* in the treatment of Ca Ski human cervical cancer cells, it was thus necessary to further expand this area of research to other cancer cell lines. According to Malaysian Cancer Statistics [13], colorectal cancer is one of the leading cancers in Malaysia and a total of 2,866 cancer cases were diagnosed among Malaysians in Peninsular Malaysia in the year 2006. In this study, we evaluated the cytotoxic activity of the extracts against three colon cancer cell lines with varying molecular characteristics, HT-29 (APC, type II truncation and COX-2 constitutive expression), HCT-15 (COX-2 deficient) and HCT 116 (APC, wild-type and COX-2 inducible) [14]. The resulting information will certainly provide scientific support upon the traditional usage of *L. indica*.

## Methods

### Chemicals and reagents

Gallic acid, BHA (butylated hydroxyanisole), ascorbic acid, DPPH (1,1-diphenyl-2-picrylhydrazyl), potassium ferricyanide, Folin-Ciocalteu’s phenol reagent, MTT [3-(4,5-dimethylthiazol-2-yl)-2,5-diphenyltetrazolium bromide], RPMI 1640 medium and McCoy’s 5A medium were obtained from Sigma-Aldrich Company. Trichloroacetic acid, ferric chloride, ethanol, hexane and ethyl acetate were purchased from Merck Company. Foetal bovine serum, penicillin, streptomycin and fungizone were from PAA Lab (Austria). SOD (superoxide dismutase) kit was purchased from Sigma-Aldrich Company.

### Plant sample collection and identification

The fresh leaves of *L. indica* were collected from Seremban, Negeri Sembilan, Malaysia in February 2011. The plants were identified by Dr Yong Kien Thai of Institute of Biological Sciences, Faculty of Science, University of Malaya, Malaysia and a voucher specimen (herbarium no: KLU47724) was deposited at the herbarium of the Institute of Biological Sciences, Faculty of Science, University of Malaya, Kuala Lumpur, Malaysia.

### Preparation of extracts

The extracts were prepared as previously described [15]. Briefly, the leaves of *L. indica* (2.70 kg) were washed, dried (38°C) and ground to fine powder (1.60 kg, 59.26%). The dried, ground leaves (300.30 g) were extracted with ethanol (3x 1.5 L) at room temperature yielding a dark green crude ethanol extract (27.80 g, 9.26%). The ethanol extract (24.80 g) was further extracted with hexane to give a hexane-soluble extract (5.80 g, 23.39%) and a hexane insoluble residue. The hexane-insoluble residue was further partitioned between ethyl acetate–water (1:1, 100 ml: 100 ml) to give an ethyl acetate-soluble extract (3.60 g, 14.52%). The water layer was freeze-dried to give a brown coloured fractionated water extract (3.60 g, 14.52%). All the extracts (ethanol, hexane, ethyl acetate and water) were kept in the dark at 4°C for not more than one week prior to evaluation of total phenolic content, antioxidant effect and cytotoxicity.

### Determination of total phenolic content

The concentrations of phenolic compounds in the extracts of *L. indica* leaves were measured according to the Folin-Ciocalteu method as previously described [16]. Briefly, extract solution (0.02 ml) at different concentrations (concentrations ranging from 0 to 20 mg/ml) was mixed with 1.58 ml of distilled water. Folin-Ciocalteu’s phenol reagent (0.1 ml) was then added to each test tube. After 3 min, 0.3 ml of saturated sodium carbonate solution was added to the mixture. The reaction mixtures were incubated in dark at 40°C for 30 min. The absorbance was measured at 765 nm with a spectrophotometer. All extracts were assayed in triplicate. Gallic acid solutions with concentrations ranging from 25 to 1000 mg/l were used for calibration. A dose response linear regression was generated by using the gallic acid standard absorbance and the levels in the samples were expressed as gallic acid equivalents (mg of GAEs/g of extract).

### Scavenging activity on 1,1-diphenyl-2-picrylhydrazyl (DPPH) radicals

The scavenging activity of the extracts of *L. indica* on DPPH radicals was measured according to the method as previously described [16]. Briefly, extract solution with different concentrations (concentrations ranging from 0 to 5 mg/ml) was mixed with 0.8% of DPPH solution. The reaction mixtures were incubated at room temperature and allowed to react for 30 minutes in the dark. All measurements were done in dim light. The absorbance was measured at 520 nm with a spectrophotometer. All assays were conducted in triplicate. The scavenging activity (%) on DPPH radical was calculated according to the following equation:

Scavenging activity (%) = [(A_control_-A_sample_)/A_control_] x 100%; where A_control_ is the absorbance of the control and A_sample_ is the absorbance of the tested extract.

The scavenging ability of the extracts was expressed as EC_50_ value, which is the effective concentration at which 50% of DPPH radicals were scavenged. The EC_50_ value was obtained from the graph of scavenging activity (%) versus concentration of samples. Ascorbic acid was used as positive reference standard.

### Reducing power assay

The reducing power of the prepared extracts was determined according to method as previously described [16]. Briefly, extract solution at different concentrations (concentrations ranging from 0 to 0.8 mg/ml) was added with 2.5 ml of 0.2 M phosphate buffer (pH 6.6) and 2.5 ml of 1% (w/v) solution of potassium ferricyanide. The mixture was incubated in a water bath at 50°C for 20 min. Following this, 2.5 ml of 10% (w/v) trichloroacetic acid solution was added and the mixture was then centrifuged at 1000 rpm for 10 min. A 2.5 ml aliquot of the upper layer was combined with 2.5 ml of distilled water and 0.5 ml of a 0.1% (w/v) solution of ferric chloride. The absorbance was measured at 700 nm with a spectrophotometer. All assays were conducted in triplicate. Ascorbic acid was used as positive reference standard.

### Detection of superoxide dismutase (SOD) activity

SOD activity was measured using water-soluble tetrazolium salt (WST) according to the method described by [17]. This method utilizes Dojindo’s WST-1, which can produce a water soluble formazan dye upon reduction with superoxide anion. After addition of all the working solution and extract solution with different concentrations (concentrations ranging from 0 to 20 mg/ml) in each well as described in the SOD kit manual, the ninety-six-well microplate was agitated and incubated at 37°C for 20 min. Absorbance was taken using microplate reader (Oasys UVM340) at 450 nm. Percentage inhibition of each sample was calculated by using following equation: {[(B1 – B3) - (S – B2)]/(B1 – B3)} x 100 where B1, B2, B3 and S were the absorbance at 450 nm for Blank 1, Blank 2, Blank 3 and sample, respectively. BHA was used as positive reference standard.

### Cell lines and culture medium

The colon cancer cell lines HT-29, HCT-15 and HCT-116 were purchased from American Type Culture Collection (ATCC, USA). The HCT-15 cells were maintained in RPMI 1640 medium; HCT 116 and HT-29 cells in McCoy’s 5A medium, supplemented with 10% foetal bovine serum, 2% penicillin or streptomycin and 1% of fungizone. The cells were cultured in a 5% CO_2_ incubator (Shel Lab water-jacketed) kept at 37°C in a humidified atmosphere.

### MTT [3-(4,5-dimethylthiazol-2-yl)-2,5-diphenyltetrazolium bromide] assay

The cytotoxic activities of samples were evaluated using MTT assay according to the method described by Mosmann [18]. Cytotoxicity of each extract was expressed as IC_50_ value, which is the concentration of extract that reduced the viability of the cells by 50% compared to the control, which were treated with 0.5% DMSO. Three replicate plates were performed for each sample. *Cis*-platin was used as positive reference standard.

### Statistical analysis

The antioxidant data in the present study were subjected to one-way analysis of variance (ANOVA) and the significance of the difference between the means was determined by the Duncan’s multiple range tests at 95% least significant difference (p < 0.05). The Pearson correlation analysis was performed to determine the correlation between total phenolic content and antioxidant activity of the extracts. Statistical significance was set at p < 0.05. The IC_50_ values for cytotoxic activity were obtained by non-linear regression using GraphPad Prism statistical software.

## Results and discussion

### Total phenolic content of *L. indica* leaf extracts

The antioxidant activity of phenolics (such as phenolic acids, flavonoids and tannins) is mainly due to their redox properties, which allow them to act as reducing agents, hydrogen donators, and singlet oxygen quenchers [19]. The Folin-Ciocalteu method is a routine assay in studying phenolic antioxidants as it is rapid, convenient, simple and reproducible. In the present study, the absorbance value of the *L. indica* extract after subtraction of control (y) was translated into total phenolic content [mg/l of gallic acid equivalents (GAEs)] using the gallic acid calibration plot with the following formula:

(1) $$\text{Total phenolic content =(y +}\;0\text{.0272)/}0{\text{.0009; R}}^{2}=0.9959$$

The quantification for total phenolic content in the *L. indica* leaf extracts, expressed as mg of GAEs/g of extract is shown in Table1. All extracts contained a considerable amount of phenolic metabolites from 1.27 to 37.29 mg of GAEs/g of extract. The highest amount was found in the fractionated water extract (37.29 mg of GAEs/g of extract), followed by ethanol (19.15 mg of GAEs/g of extract), ethyl acetate (15.61 mg of GAEs/g of extract) and hexane (1.27 mg of GAEs/g of extract) extracts in the decreasing order. The hexane extract showed the lowest phenolic content although the yield of hexane extract was the highest among the fractionated extracts (refer to the section ‘preparation of extracts’). The significantly higher (p < 0.05) phenolic content in the fractionated water extract than in the crude ethanol extract was probably due to the concentration of phenolic compounds in this fractionated extract. In the previous study reported by Srinivasan et al. [6], gallic acid which is a strong naturally occurring antioxidant was identified in the leaf extract. The high phenolic content in the fractionated water extract might contribute towards the antioxidant activities and curative ability adsorbing and neutralising free radicals. 

**Table 1:** **Total phenolic content of *L. indica *extracts**

**Extracts**	**Concentration of total phenolics (mg of GAEs/g of extract)**
Ethanol	19.15 ± 2.66^c^
Hexane	1.27 ± 0.09^a^
Ethyl acetate	15.61 ± 2.12^b^
Water	37.29 ± 3.52^d^
Ascorbic acid*	45.03 ± 2.15^e^

*Positive reference standard; GAEs, gallic acid equivalents; Values are expressed as mean ± standard deviation (n = 3), means with different letters (a-e) in the same column were significantly different (p < 0.05, ANOVA).

### Antioxidant activities of *L. indica* leaf extracts

There are various published methods measuring the antioxidant capacity *in vitro*, but no single assay is capable to determine the total antioxidant ability of a studied sample. Previous studies [20,21] indicated that more than one antioxidant capacity measurement is needed in order to take into account the various modes of antioxidants’ actions. Thus, the antioxidant activity of *L. indica* leaf extracts were evaluated by employing three different established testing systems, such as scavenging activity on DPPH radicals, reducing power assay and SOD activity assay. These three assays have been widely used to assess the antioxidant abilities of studied sample as they required relatively standard equipment and reproducible results.

### Scavenging activity of *L. indica* leaf extracts on DPPH radicals

Free radical scavenging is one of the known mechanisms by which antioxidants inhibit lipid oxidation. DPPH is a nitrogen-centered free radical which stable in room temperature. The reducing capability of the DPPH is determined by the reduction in its absorbance measured at 520 nm induced by antioxidants. The decrease in the absorbance of DPPH caused by antioxidants is due to the reaction between antioxidant molecules and radical, which results in the scavenging of the radical by electron transfer.

In the DPPH scavenging assay, leaf extracts of *L. indica* were investigated through the free radical scavenging activity via their reaction with the stable DPPH radicals. The radical scavenging activity (EC_50_) values of the extracts are shown in Table2. Low EC_50_ value indicates strong ability of the extract to act as DPPH scavenger. A high EC_50_ value indicates low scavenging activity of the scavengers as more amounts of the scavengers were required to achieve 50% scavenging reaction. This means, the scavengers are less effective in scavenging the DPPH radicals. Among the four extracts, the fractionated water extract (EC_50_ 48 μg/ml) showed the strongest scavenging activity compared with the standard ascorbic acid, followed by ethanol (EC_50_ 60 μg/ml) and ethyl acetate (EC_50_ 68 μg/ml) extracts. The hexane extract showed the weakest scavenging activity on DPPH radicals with the EC_50_ value of 1285 μg/ml. The present result is consistent with the previous data reported by Saha et al. [8], which showed that methanol extract from the whole plant of *L. indica* also showed strong free radical scavenging activity comparable with quercetin, BHT (2,6-di-*t*-butyl-4-methylphenol) and Vitamin C. 

**Table 2:** **The scavenging activity (EC**_**50**_**values) of *L. indica *extracts on DPPH radicals**

**Extracts**	**EC**_**50**_**values (μg/ml)**
Ethanol	60
Hexane	1285
Ethyl acetate	68
Water	48
Ascorbic acid*	15

*Positive reference standard.

The results in Table1 and Table2 show that there is a positive correlation between higher total phenolic content in the extracts and stronger DPPH scavenging activity. This conclusion is supported by published reports which indicated that phenolic substances generally well-correlated with scavenging activity on DPPH radicals [22,23]. Hence, the strong DPPH scavenging activity of fractionated water extract (Table2) may be attributed by the high content of phenolic compounds (Table1), although other antioxidants may present in the fractionated water extract as well.

### Reducing power of *L. indica* leaf extracts

Previous studies have pointed out that the antioxidant capability is always related to the development of reductones which can terminate the free radical chain reactions [24-26]. The reducing power of *L. indica* extracts are shown in Table3. In the reducing power assay, a stronger absorbance indicates a higher reducing power. The extracts that showed comparable absorbance readings with the positive reference standard are considered to have high reducing power. 

**Table 3:** **Reducing power of *L. indica *extracts at various concentrations**

**Extracts**	**Concentrations of extracts (mg/ml)**			
	**0.2**	**0.4**	**0.6**	**0.8**
Ethanol	0.72 ± 0.02^cw^	1.26 ± 0.02^cx^	1.85 ± 0.07^dy^	2.07 ± 0.05^cz^
Hexane	0.11 ± 0.03^aw^	0.20 ± 0.01^ax^	0.23 ± 0.01^ax^	0.30 ± 0.01^ay^
Ethyl acetate	0.57 ± 0.02^bw^	1.07 ± 0.03^bx^	1.27 ± 0.04^by^	1.52 ± 0.06^bz^
Water	0.91 ± 0.03^dw^	1.37 ± 0.03^dx^	1.76 ± 0.02^cy^	2.70 ± 0.02^dz^
Ascorbic acid*	1.84 ± 0.04^ew^	2.59 ± 0.04^ex^	2.65 ± 0.02^ey^	2.73 ± 0.03^dz^

* Positive reference standard; Values expressed are mean ± standard deviation (n = 3). For different extracts with the same concentration, means in the same column with different letters (a-e) were significantly different (p < 0.05, ANOVA). For the same extract or standard with different concentrations, means in the same row with different letters (w-z) were significantly different (p < 0.05, ANOVA).

**Figure 1:**
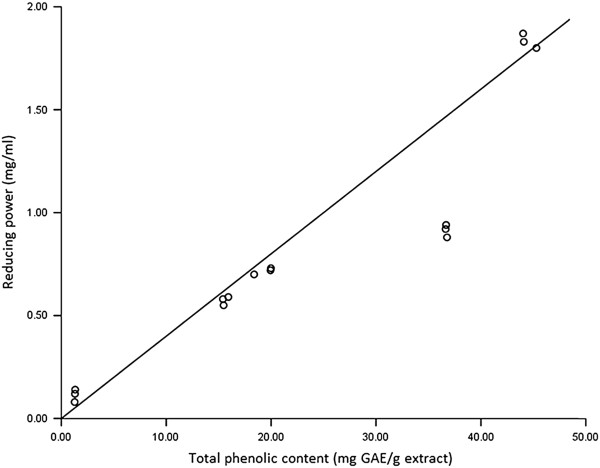
**Correlation between total phenolic content and reducing power of L. indica leaf extracts.** Correlation coefficient r = 0.928 (p > 0.01).

The reducing power of the four extracts varied significantly (p < 0.05) at different concentrations (Table3). The reducing power of all the extracts increased gradually with the increase in concentrations of the extracts. Generally, the fractionated water extract showed a significantly (p < 0.05) higher reducing power at all the tested concentrations compared to the other extracts. This was followed by ethanol and ethyl acetate extracts, while the hexane extract showed the lowest reducing power among all the tested concentrations. At concentration 0.8 mg/ml, the reducing power of fractionated water extract was comparable to that of ascorbic acid. A strong correlation (r = 0.928) was found between total amount of phenols and reducing power of *L. indica* extracts (Figure1), indicating that the phenol compounds play an important role in the reducing power of the extracts. This finding is in agreement with previous publication by Krishnaiah et al. [27]. 

### Superoxide dismutase (SOD) activity of *L. indica* leaf extracts

Superoxide dismutase (SOD) which catalyzes the dismutation of superoxide anion into hydrogen peroxide and molecular oxygen is one of the most important antioxidative enzymes [28]. In the present study, a sensitive water-soluble tetrazolium salt, WST-1 [2-(4-iodophenyl)-3-(4-nitrophenyl)-5-(2,4-disulfophenyl)-2 H-tetrazolium, monosodium salt] was used in the SOD assay. WST-1 produces a high water-soluble formazan dye upon reduction with superoxide anion. The rate of the reduction with superoxide anion is linearly related to xanthine oxidase activity, and is inhibited by SOD. Thus, the inhibition activity of SOD can be determined by measuring the superoxide anion-caused formation of water-soluble dye. Higher SOD activity will reduce the formation of formazan and indirectly lower the absorbance reading as well.

The SOD activity of *L. indica* leaf extracts is shown in Table4. All the extracts showed similar levels of SOD activity (94.36-100.00% inhibition rate) with the positive reference BHA (100% inhibition rate) at highest concentration tested (20 mg/ml). The hexane, ethyl acetate and ethanol extracts possessed significantly stronger inhibition rate than positive reference BHA in SOD activity at lower concentrations tested (0.002-2.000 mg/ml). Among the *L. indica* extracts, fractionated water extract showed the strongest inhibition rate in the SOD assay significantly (p < 0.05), followed by ethyl acetate and ethanol extracts. In this assay, the hexane extract showed the lowest inhibition rate at all the tested concentrations. Strong correlation was found between the SOD activity and total phenolic content determined by Folin-Ciocalteu method (Table1). This result is in agreement with previous report that indicates SOD activity can be related to phenolic content [29]. 

**Table 4:** **Inhibition rate of *L. indica *leaf extracts measured by SOD assay**

**Extracts**	**Concentrations of extracts (mg/ml)**				
	**0.002**	**0.020**	**0.200**	**2.000**	**20.000**
Ethanol	23.59 ± 1.02^bv^	70.55 ± 1.16^cw^	84.81 ± 0.81^cx^	90.85 ± 4.70^cy^	94.36 ± 3.26^ay^
Hexane	0.78 ± 0.46^av^	1.21 ± 0.42^av^	17.17 ± 0.72^aw^	70.86 ± 4.06^ax^	100.00 ± 4.55^by^
Ethyl acetate	32.35 ± 2.52^cv^	78.98 ± 2.69^dw^	99.02 ± 1.40^dx^	100.00 ± 2.27^dx^	98.37 ± 1.97^bx^
Water	40.52 ± 3.59^dv^	88.13 ± 2.88^ew^	98.80 ± 1.80^dx^	100.00 ± 1.45^dx^	100.00 ± 4.14^bx^
BHA*	0.00 ± 0.79^av^	15.41 ± 0.16^bw^	54.67 ± 0.76^bx^	81.42 ± 3.44^by^	100.00 ± 4.22^bz^

* Positive reference standard; Values expressed are mean ± standard deviation (n = 3). For different extracts with the same concentration, means in the same column with different letters (a-e) were significantly different (p < 0.05, ANOVA). For the same extract or standard with different concentrations, means in the same row with different letters (v-z) were significantly different (p < 0.05, ANOVA).

### *In vitro* cytotoxic activity of *L. indica* extracts

The cytotoxic activity of the *L. indica* leaf extracts against three colon cancer cell lines with varying molecular characteristics, HT-29 (APC, type II truncation and COX-2 constitutive expression), HCT-15 (COX-2 deficient) and HCT 116 (APC, wild-type and COX-2 inducible) were evaluated by MTT assay in the present study. MTT assay measures the cell viability based on the reduction of yellow tetrazolium MTT to purple formazon dye by mitochondrial dehydrogenase enzyme. The amount of formazon reflects the number of metabolically active viable cells [30]. According to the United States National Cancer Institute plant screening program, a plant extract is generally considered to have active cytotoxic effect if the IC_50_ value is 20 μg/ml or less, after incubation between 48 to 72 hours [31]. Table5 shows the results of cytotoxicity screening of *L. indica* leaf extracts, expressed as IC_50_ values, averaged from three experiments. All the four extracts of *L. indica* leaf extracts did not show cytotoxic effects against the three tested human colon cancer cell lines (IC_50_ ≥ 100 μg/ml in all cases) after incubation for 72 hours. According to the published data reported by Nurhanan et al. [10], the methanol extracts of *L. indica* did not exert any cytotoxicity against the MCF-7 and T47D breast cancer cell lines (IC_50_ > 100 μg/ml in both cell lines). 

**Table 5:** **Cytotoxic activity (IC**_**50**_**values) of *L. indica *extracts against three human colon cancer cell lines**

**Extracts**	**IC**_**50**_**(μg/ml)**		
	**HT-29**	**HCT-15**	**HCT-116**
Methanol	>100	>100	>100
Hexane	>100	>100	>100
Acetyl acetate	>100	>100	100.0
Water	>100	>100	>100
*Cis*-platin*	6.4	1.7	2.9

*Positive reference standard.

## Conclusions

This study was designed to investigate the phenolic content, antioxidant effect and cytotoxic activity of *L. indica* leaf extracts. The antioxidant activity of the extracts correlated well with the total phenolic contents and indicated that phenolic compounds are dominant contributors to the antioxidant activity of the extracts. This finding is supported by published manuscript [32] which indicates that phenolic compounds have the abilities to quench lipid peroxidation, prevent DNA oxidative damage and scavenge the reactive oxygen species. Overall, the fractionated water extract of *L. indica* leaves which contained the highest amount of phenolic compounds, exhibited outstanding reducing power, strong DPPH radical scavenging activity and pronounced inhibition rate in SOD assay. All the four extracts exert no damage to the selected colon cancer cells (HT-29, HCT-15 and HCT-116) in the MTT assay.

The data obtained in these testing systems clearly establish the antioxidant potency of the fractionated water extract of *L. indica* leaves. Future studies should be carried out to identify the active compounds in the fractionated water extract, in order to provide more convincing evidence. An investigation into this phenomenon is now underway.

## Competing interests

The authors declare that they have no competing interests.

## Authors’ contributions

RNS prepared the extracts and carried out the total phenolic content as well as the antioxidant studies. NS worked on the cytotoxicity screening. SSK co-worked on antioxidant assays and analyzed the data for antioxidant assays. WNA evaluated the data and edited the manuscript. SKS designed the current project, supervised the work and wrote the manuscript. All authors have read and approved the final manuscript.

## Pre-publication history

The pre-publication history for this paper can be accessed here:

http://www.biomedcentral.com/1472-6882/12/128/prepub

## Author details

^1^Institutional address: Institute of Biological Sciences, Faculty of Science, University of Malaya, 50603 Kuala Lumpur, Malaysia. ^2^Institutional address: Biology Division, Centre for Foundation Studies In Science, University of Malaya, 50603 Kuala Lumpur, Malaysia.
